# Physiological processes and gross energy budget of the submerged longline-cultured Pacific oyster *Crassostrea gigas* in a temperate bay of Korea

**DOI:** 10.1371/journal.pone.0199752

**Published:** 2018-07-05

**Authors:** Young-Jae Lee, Eunah Han, Michael J. Wilberg, Won Chan Lee, Kwang-Sik Choi, Chang-Keun Kang

**Affiliations:** 1 School of Earth Sciences and Environmental Engineering, Gwangju Institute of Science and Technology, Gwangju, Republic of Korea; 2 Chesapeake Biological Laboratory, University of Maryland Center for Environmental Science, Solomons, Maryland, United States of America; 3 Marine Environment Research Division, National Institute of Fisheries Science, Busan, Republic of Korea; 4 Faculty of Marine Biomedical Science, Jeju National University, Jeju, Republic of Korea; University of Hong Kong, HONG KONG

## Abstract

Physiological processes and gross energy budget of the longline-cultured Pacific oyster *Crassostrea gigas* were investigated in Geoje–Hansan Bay, Korea during two entire culturing periods. Based on physiological measurements of food consumption, feces production, ammonium excretion, and respiration from July 2008 to February 2009 and from July 2013 to February 2014, scope for growth appeared to be positive during most of the culturing period, except for one period with extremely high temperatures (up to 25°C). Estimates of physiological energy production matched well with tissue energy increment measured by gross biochemical composition during the culturing period, suggesting that the oysters might adjust their physiological performance to relatively low concentrations of suspended particulate matter in the bay to optimize energy acquisition. Such an adaptive adjustment includes an increased absorption of energy and a reduced loss of metabolic and excretory energy, resulting in positive production under high culturing density. Using physiological measurements, we further assessed the feedback effects of the longline aquaculture of oysters on the bay system. Ecological efficiency, estimated by a series of energetic efficiencies at the whole bay level, was low compared with Lindeman’s law of trophic efficiency. Biodeposition and ammonia excretion rates in this study were relatively low compared with other intertidal plastic bag cultures. These results indicate that the cultured oysters might have only minor effects on benthic and pelagic environments of the bay. Overall, our results suggest that the adaptive physiological performance of oysters and consequently weak feedback effects on ambient habitats should facilitate sustainable longline aquaculture in the bay for a prolonged period without severe habitat deterioration.

## Introduction

Because of its high productivity and wide-range of environmental tolerance, the Pacific oyster *Crassostrea gigas* has been introduced to many countries for aquaculture [1,2]. This species is relatively easy to control for this purpose and does not need the supply of additional food for somatic or gametogenic growth. Its ability to adapt to various environmental conditions, coupled with its’ rapid growth and low mortality, enables to flourish oyster culture [3]. Like cultured bivalves such as mussels, scallops, and clams, there are potential benefits of oyster aquaculture due to high harvesting weight and short growth cycles with ecological and commercial considerations [4]. In this respect, as defined by four functional categories (physical, production, ecological, and social carrying capacities), attempts have been made to evaluate the optimal stocking density of a given culturing area to satisfy these highly interlinked considerations and facilitate sustainable development of oyster aquaculture [5–8]. Because optimal stocking density is difficult to evaluate simply with in situ measurements, a wide range of numerical modeling approaches has been applied to assess the carrying capacity of an area, thereby addressing different scenarios regarding the effects of varying environmental conditions on bivalve growth and vice versa [4,7]. Considering that in many cases, however, information on bivalve feeding and physiology is acquired from studies conducted elsewhere, the realistic growth dynamics of oysters under varying environmental conditions and feedback mechanisms between the culture activities and the ecosystem in an area of interest should be addressed by field experiments.

Food consumption, feces production, ammonia excretion, and respiration rates of oysters can provide information on their ecophysiological mechanisms to optimize energy gain in response to varying environmental conditions [9,10]. The energy budgets of individual organisms can be completed by the physiological adjustments and reflected in the scope for growth (SFG, i.e., instantaneous production immediately after food assimilation; [9]). As a result, tissue growth of oysters during an entire culturing period represents a time-integrated response of physiological activities, i.e., the gross energy budget [11,12,13]. The resultant surplus energy during growth is stored in the form of biochemical constituents (i.e., energy reserves) and expended on metabolic demand for maintenance and gametogenesis [14,15,16]. In this context, the cultured oysters filter particles from the water column and produce fecal pellets that can enhance benthic–pelagic coupling [17,18]. Indeed, individual physiological activities can regulate the removal of phytoplankton and organic debris such as detritus from water columns [19,20], and biodeposition [11,21,22] and excretion may modulate nitrogen release [19,23] to the environment, including sediments and water columns, contributing to their roles as ‘ecosystem engineers’ [24,25,26].

Environmental conditions, such as water temperature and food availability, determine physiological functions in culturing oysters [9]. These environmental conditions in the coastal waters off the Korean peninsula are characterized by a unique seasonality with the northeast Asian monsoon. Water temperature of this location displays the typical variation of temperate zones, being highest in summer [27,28]. Phytoplankton biomass (also primary productivity), which determines food availability for the oysters, peaks in summer in response to the concentrated nutrient inflow via freshwater discharge during the summer monsoon [28,29,30]. The cultured oysters are thus expected to experience seasonal changes in their energy balance being responsive to the environmental variability of this location, and their actual growth performance will reflect the gross energy budget during the culturing period. On the southern coast of Korea, the oyster spats are usually suspended on ropes using longlines in water columns of subtidal grow-out areas in early summer, and the adults are harvested in winter. An energetic description of growth is needed to evaluate whether the traditional oyster-culturing strategy optimizes their growth performance depending on the typical environmental variability of this locale. Furthermore, measurements of individual physiological activities might enable the assessment of potential feedback effects caused by the high culturing density of oysters in culturing environments [11,12,31].

In the present study, physiological energetics and gross biochemical composition of *C*. *gigas*, cultured on ropes using suspended longlines in a semi-closed bay system, Geoje–Hansan Bay of Korea, were measured monthly under the field conditions during an entire culturing period. The objectives were: (1) to assess the physiological responses of oysters including food consumption, feces production, ammonia excretion, and respiration) to field culture conditions and correspondingly temporal patterns of energy budget (i.e., SFG); (2) to determine their energy balance-related patterns of reserve storage and growth; and (3) to establish a gross energy budget for typical individuals of mean tissue weight during an entire culturing period. Finally, an additional attempt to construct an overall energy budget of entire cultured oyster stocks within the bay during the culturing cycle was made by using the stock density (i.e., the numbers of individuals) of cultured oysters.

## Materials and methods

### Study site

The study was carried out in Geoje–Hansan Bay on the southern coast of Korea (34° 80′ N and 128° 55′ E). Historically, the first attempt to culture oysters using suspended longlines in Korea was made in the bay in the late 1960s, and other suspension-feeding species such as mussels and tunicates are now being cultured in a similar manner. The bay is semi-closed with a relatively large area of about 55 km^2^ (about 10 km long and 2–6 km wide). A total area of 6.6 km^2^ within the bay, corresponding to 12% of the bay area, is used for oyster cultivation [32]. Annual oyster production was recorded at about 6383 t for wet tissue weight in 2014 [33]. Water depth ranges from 6.5 m in the inner bay to 27.9 m in the outer water channel. Monthly mean water temperatures vary from 9.0 to 25.6°C at the surface layer and from 9.0 to 21.1°C at the bottom layer [34]. Monthly mean salinities fall within very narrow ranges of 29.5 to 34.2 at the surface layer and 32.8 to 34.2 at the bottom layer. Monthly mean chlorophyll *a* concentrations display a great seasonal fluctuation of 1.33–18.0 μg l^−1^. Concentrations of dissolved oxygen are high with 7.7–10.4 mg l^−1^ in the surface layer and 4.4–10.0 mg l^−1^ in the bottom layer throughout the year; hypoxia (<5 mg l^−1^) can be observed in the bottom layer in a very limited area of the innermost part of the bay. Flow speeds on spring tides are 20–60 cm s^−1^ in the outer bay channel and 5–15 cm s^−1^ in the inner bay area on both the ebb and flood tides, and are 10–12 cm s^−1^ in the channel and 2–8 cm s^−1^ in the inner bay on neap tides [35]. Consequently, residual currents display prevalent flows to the offshore from the bay in the surface layer and to the inner-bay from the offshore in the bottom layer. Accordingly, the residence time of particulate matter, which constitutes oyster food materials, is 30–65 days in the inner-bay area and 15 days in the outer-bay channel area [36]. In general, the longlines are 100 m long and stretched in parallel at intervals of 5–10 m. The vertical ropes are hung from the longline at intervals of 50–70 cm. The seed collectors, which consist of a string of about 50 scallop shells threaded on a polyethylene wire about 1 m long, are attached to the ropes at intervals of 30–50 cm. Seeds attached on the collectors are obtained from the natural seeding grounds and the hatchery. Oyster seeds collected in June–July can be moved to culturing grounds for growing out 2–3 weeks after attachment or after hardening in an intertidal zone by September. In contrast, seeds collected in August–September are hardened until the coming spring and move to a growing out system, in which 10–25 collectors are attached to a vertical rope 2–6 m long. In the bay, spat oysters generally start to grow in late spring (May–June), continue growing during summer–fall, and are harvested from November until the following February.

### Ethics statement

Sample collection and export to the sampling area were permitted by Ministry of Oceans and Fisheries of Korea. Collection and delivery of oyster specimens to a laboratory were conducted by commercial fishermen, and our field studies was excluded the usage of endangered or protected species. Institutional Animal Care and Use Committees approval for this sampling and experimental method was not required; no specific permissions were required for the location and collection because this location is commercially available for fishery.

### Experimental design for *in situ* measurement of physiological rates

Samplings and measurements of the *in situ* physiological rates of oysters were conducted monthly on a 10 × 10 m barge deployed in an oyster culturing ground during two entire culturing periods from July 2008 to February 2009 and from July 2013 to February 2014. Eighteen specimens were washed to remove epibionts immediately after samples were collected in the culturing site, and then reared in a series of 500 ml experimental chambers. Ten chambers each were used for feeding and respiration measurements. Of these, nine specimens were placed individually in flow-through open cylindrical chambers (i.e., feeding chambers), which were designed to determine food consumption, feces production, and ammonium excretion rates. The remaining nine specimens were also individually placed in the flow-through closed chambers, each equipped with a magnetic stirrer to measure respiration rates. For both measurements, one chamber was used as a control without an oyster. Each series of chambers were partially or absolutely immersed, respectively, in large aquaria (50 × 150 × 50 cm), which were filled with local seawater to maintain natural environmental conditions. Seawater was pumped from 1 m below the sea surface and supplied continuously into individual experimental chambers from a main mixing tank using a peristaltic pump equipped with a 10-channel pump head (BT 100-1L, Baoding Longer Precision Pump Co. Ltd, Baoding, P. R. China). Flow rates of experimental chambers were set between 30 and 50 ml min^−1^ depending on an oyster size. These allowed the proportion of particles in the outflow of the feeding chamber to be maintained at over 80% of the inflow [37] and the concentrations of dissolved oxygen in the experimental chamber to be over 80% of *in situ* concentrations [38]. All experimental specimens were acclimated for 6 h in the experimental chamber conditions, and then all experiments were performed for 24 h.

After evacuation of the gut contents of specimens into filtered seawater following the experiments, they were stored in an ice box containing dry ice and transported to the laboratory. Specimens were then dissected carefully with a stainless steel knife. The shells of specimens were dried in a drying oven for about 72 h and then weighed to determine their dry shell weight (SW). Flesh tissues were lyophilized for 72 h, weighed to determine dry tissue weight (DW), and then pulverized for subsequent biochemical analysis as described below (see Section 2.4).

### Experimental conditions and physiological measurements

For each experiment, water temperature was recorded every 1 h using a CTD meter (Sea-Bird Electronics Inc., Bellevue, WA, USA). To determine food conditions during each experiment, suspended particulate matter (SPM) concentrations were determined every 4 h over a 24-h period and converted into energy equivalent by biochemical analyses (i.e., contents of proteins, carbohydrates, and lipids). Water samples were pre-filtered through a 180 μm Nitex mesh to remove any zooplankton and large particle debris and stored in acid-washed plastic bottles on ice. The samples was filtered onto pre-combusted (450°C for 4h) and pre-weighed Whatman GF/F filters (47 mm, 0.7 μm pore size). The filters were than lyophilized for 72 h and weighed to determined SPM concentrations. Biochemical compositions of SPM were measured, and then the amount of food energy supplied for individual experimental chambers were quantified by converting biochemical components into their energy equivalents by the procedure described below (Section 2.4). Chlorophyll *a* was extracted in 90% acetone for 24 h in the dark at ‒20°C, and then measured using a fluorometer (Turner Designs, Sunnyvale, CA, USA) according to the method of Holm-Hassen [39].

Four physiological components were measured to estimate the monthly SFG of cultivated oysters. The food consumption rate (C) was calculated from the reduction of SPM between the outflows from the control chamber and each experimental chamber containing individual specimens, which indicates a measure of the removal of SPM by individual oysters. During each experiment, water samples were collected every 4 h over a 24-h period from the outflows of each feeding chamber, and their SPM concentrations and biochemical components were determined using the same procedures as described above for measuring experimental conditions. Finally, the rate was converted into energy equivalents (J d^−1^) by the procedure described below (Section 2.4).

The fecal production rate (F) of individual oysters was estimated by measuring the energy values of feces directly collected every 2 h over a 24-h period from each experimental feeding chamber. Fecal materials collected with a Pasteur pipette were placed into a 5 ml pre-combusted and pre-weighed glass tube, rinsed with Milli-Q-water to eliminate salt contents, lyophilized, pulverized, and weighed. Their biochemical components (i.e., proteins, carbohydrates, and lipids) were analyzed using the methods described below (Section 2.4). The F values were also converted into energy equivalents (J d^−1^).

Ammonia excretion rate (U) was determined by the increment in ammonia concentration between the outflows of the control and the individual experimental chambers. Ammonia analysis was conducted immediately after water samples were collected when SPM was collected during each experiment. Ammonia concentration was determined by the colorimetric method [40]. Next, the rate was calculated by converting the amounts increased into energy equivalents using a conversion factor of 24.83 J mg^−1^ NH_4_-N [41].

Respiration rate (R) was measured by the difference in oxygen concentrations between the control and the experimentally closed flow-through chambers. Dissolved oxygen concentration was continuously measured inside the measurement chambers using oxymetric probes (Oxy-10 micro, PreSens-Precision Sensing GmbH, Regensburg, Germany) for 24 h. Detailed procedures of the continuous measurement and calculation of oxygen consumption have been described in [42]. The conversion factor of 1 mg O_2_ = 14.0 J was applied to converting the oxygen consumption rate into an energy equivalent (J d^−1^) [43].

The SFG, which represents the energy available for somatic and gametogenic growth, was calculated using the energy budget equation in an individual organism as follows [9]: C = SFG + F + U + R; SFG = C–(F + U + R) = A–(U + R), where C = consumption, F = feces production, U = ammonia excretion, A = absorption, and R = respiration. No individuals stopped their physiological activities during the measurements. Although a few specimens paused shortly for respiration, it was ignored in the respiration rate calculations. Then, net growth efficiency (*K*_2_) was calculated following the formula [9]: *K*_2_ = SFG / A.

### Biochemical analysis

Aliquots of 5–10 mg powdered tissue samples of 18 individuals per month were analyzed to determine their biochemical constituents of proteins, carbohydrates, and lipids. Total proteins were determined colorimetrically with bovine serum albumin as a standard using the Folin–Ciocalteu phenol method [44] after alkaline hydrolysis with 1 N NaOH at 60°C for 30 min. Total carbohydrates were extracted with 15% trichloroacetic acid, and only glycogen was precipitated by centrifugation (2000 *g*) with 99% ethanol for 10 min. Carbohydrates and glycogen were determined using the phenol–sulfuric acid method [45] with glucose as a standard. Lipids were extracted using a mixture of chloroform and methanol (1:2) [46], and total lipids were determined with glyceryl tripalmitate as a standard using the spectrophotometric method [47]. Energy equivalents of proteins, carbohydrates, and lipids were estimated using the conversion factors 24.0, 17.5, and 39.5 J mg^−1^, respectively [43].

### Standardization of biochemical and physiological measurements

Because most physiological and biochemical functions of organisms vary allometrically with their body size, standardization to a particular body size is necessary to eliminate the confounding effects of variation in body size and thereby evaluate their physiological states independent of growth [48]. Here, biochemical, physiological, and biometric variables on each sampling occasion were fitted to the allometric equation, *Y* = *aW*^*b*^, where *Y* is the variable of interest, *W* is dry SW or DW, and *a* and *b* are the fitted constants. Least-squares regression analyses between the variables of interest and biometric components (i.e., SW or DW) following logarithmic transformation (base 10) of all variables were then performed to determine the fitted constants representing the intercepts and slopes of the regression equations. Analysis of covariance (ANCOVA) was subsequently employed to test the significance of any differences in slopes among regression equations that were obtained monthly [49].

When there was no significant difference between slopes, a common slope and the adjusted intercepts were calculated, and used to estimate DWs of monthly mean-sized SW individuals (hereafter, monthly mean DWs). When there was a significant difference between slopes, DWs of monthly mean-sized SW individuals were computed by substituting monthly mean values of SW in the monthly fitted original slopes and intercepts (S1 Table). In the same manner, gross weights of the biochemical constituents were then related to DW. Because of significant differences between estimates of the slope for protein and glycogen, these were computed for the monthly mean SW by substituting the appropriate values of DW on each sampling occasion in the monthly fitted original equations (S2 Table). However, because there was no significant difference between slopes for lipid and carbohydrates, a common slope and the adjusted intercepts were calculated. The gross weights of the biochemical constituents were then expressed as mg per monthly mean-size individual.

Because the ANCOVA revealed no significant differences among the estimates of slopes among monthly regressions between physiological rates (i.e., consumption, feces production, ammonia excretion, and respiration) and DW of the oysters, a common slope ($\bar{b}$) and the intercepts (*ā*) for each physiological variable were re-calculated. A Bonferroni *post hoc* multiple comparison test was conducted to test differences between intercepts. Individual physiological rates were recalculated, and then used to calculate physiological rates of monthly mean-DW individuals using the common slopes ($\bar{b}$) and intercepts (*ā*), before being expressed as monthly mean physiological rates in the present study. Before applying t- tests, the data were tested for normality of distribution and equal variance, and significance was tested at P = 0.05.Statistical analyses were performed using a commercially available software package, IBM SPSS Statistics, v. 22.0 (IBM Corp., Armonk, NY, USA).

### Gross energy budget calculation

Using the common slope ($\bar{b}$) and the resultant intercepts for each physiological variable, the monthly mean values of individual physiological rates were computed by substituting the monthly mean DW values on each experiment and then expressed as J per monthly mean-sized individual. Based on those mean daily physiological rates from our monthly rate measurements, the gross energy budget for an individual of mean DW (at harvest time) during an entire culturing period was estimated. In addition, in an attempt to construct an overall energy budget for an entire culturing cycle in the whole bay area, abundance of the currently cultured oyster stocks in the bay was estimated during the study [32]. Interestingly, an extensive in situ investigation on the number of longlines and ropes deployed throughout the entire bay system, and oyster individuals per rope was conducted during the present study.

## Results

### Environmental conditions

Seawater temperatures varied between 11.2°C (January 2009) and 25.7°C (September 2008) during the first experiment during 2008−2009, and between 10.9°C (January 2014) and 22.0°C (September 2013) during the second experiment during 2013−2014 (Table 1). No significant differences in the mean seawater temperatures were found between the first and second experiments (paired *t*-test, *t*_7_ = 0.717; *P* = 0.496), except for one in September 2008 when remarkably high temperatures were observed (a mean of 25.1 ± 0.8°C). SPM fluctuated from 2.2 (July 2008) to 31.3 mg l^−1^ (November 2008) during the first experiment, and from 1.6 (October 2013) to 29.0 mg l^−1^ (January 2014) during the second experiment, showing no clear seasonal trends. No significant difference in SPM concentrations was found between the first and second experiments (paired *t*-test, *t*_7_ = −1.672; *P* = 0.138). Fraction (*f*) of particulate organic matter (%POM) of total SPM was relatively constant throughout the year with 33.9 to 49.8% (except for 15.4% in November 2008) and 25.1 to 44.9% during the first and second experiments, respectively. Chlorophyll *a* concentration ranged from 0.51 μg l^−1^ (September 2008) to 5.56 μg l^−1^ (February 2009) during the first experiment, and from 0.41 μg l^−1^ (September 2013) to 11.53 μg l^−1^ (November 2013) during the second experiment, displaying no significant difference between the two experiments (paired *t*-test, *t*_7_ = −1.282; *P* = 0.241). Food energy showed irregular seasonal variations, ranging from 3.7 (September 2008) to 16.4 J l^−1^ (February 2009) during the first experiment, and from 2.8 (October 2013) to 22.5 J l^−1^ (November 2013) during the second experiment.

**Table 1:** The range of experimental conditions.

Month	Temperature (°C)	SW (g)	DW (g)	SPM (mg l^‒1^) / *f*	Chl. *a* (μg l^‒1^)	Food energy (J l^‒1^)
July 2008	19.7–20.3	0.97–5.78	0.06–0.63	2.2–2.6 / 44.5	0.62–0.90	4.4–7.7
August	22.0–23.0	4.04–13.21	0.25–1.10	4.3–5.7 / 38.9	1.23–1.73	7.7–10.2
September	24.5–25.7	6.31–20.42	0.32–0.90	3.4–4.4 / 33.9	0.51–1.83	3.7–4.5
October	19.7–20.5	7.46–37.95	0.07–1.03	2.5–2.9 / 48.7	1.49–2.67	7.7–9.7
November	15.5–18.0	15.91–55.65	0.42–2.29	28.1–31.3 / 15.4	0.58–1.00	5.2–6.4
December	12.7–13.4	10.23–39.67	0.40–1.64	4.3–4.5 / 36.5	1.31–2.13	8.2–11.4
January 2009	11.2–11.8	19.71–48.14	0.47–2.55	2.7–2.7 / 48.7	1.28–1.58	11.8–14.2
February	11.3–11.8	16.30–63.62	0.32–2.95	6.9–7.9 / 49.8	3.16–5.56	15.1–16.4
July 2013	19.1–20.1	3.63–9.54	0.22–0.75	11.7–16.4 / 30.8	1.31–1.37	5.0–6.4
August	19.1–20.7	4.23–11.80	0.25–0.88	9.2–11.7 / 28.9	1.04–1.21	5.2–7.1
September	20.8–22.0	5.58–15.13	0.33–1.32	9.1–10.8 / 30.3	6.97–7.39	13.8–17.7
October	21.4–21.7	8.23–19.36	0.59–1.81	1.6–1.9 / 32.1	0.41–0.49	2.8–3.3
November	19.4–19.5	13.78–36.93	0.59–2.61	24.0–24.7 / 25.1	9.95–11.53	22.1–22.5
December	13.0–13.4	11.03–38.31	0.54–3.01	8.2–9.9 / 41.7	3.25–4.73	5.3–6.1
January 2014	11.3–11.7	19.69–61.81	1.06–3.79	28.7–29.0 / 32.2	1.18–1.76	19.1–19.3
February	10.9–11.1	18.55–50.34	1.56–3.93	4.9–7.2 / 44.9	2.41–2.49	8.6–9.0

Dry shell weight (SW) and dry tissue weight (DW) of experimental individuals, and composition of suspended particulate matter (SPM) supplied to *Crassostrea gigas*; *f*, fraction of percentage particulate organic matter (%POM) of total SPM; Chl. *a*, chlorophyll *a*; Food energy, energy value of SPM calculated by energy equivalents of proteins, carbohydrates, and lipids.

### Seasonal variations of SW and DW

**Fig 1:**
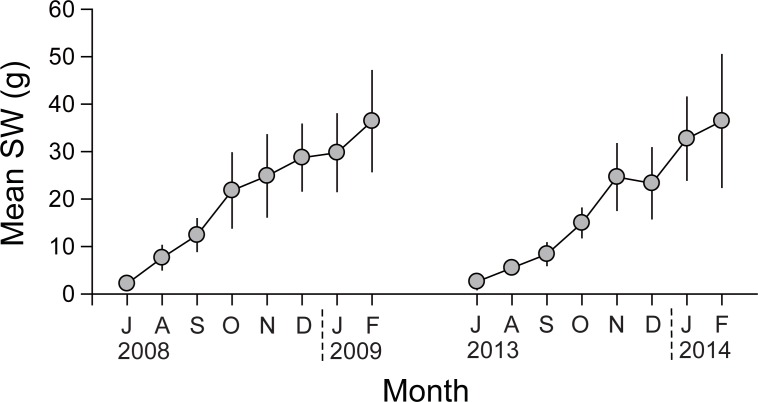
Seasonal variations in monthly mean dry shell weight (SW) of *Crassostrea gigas* in Geoje–Hansan Bay.

**Fig 2:**
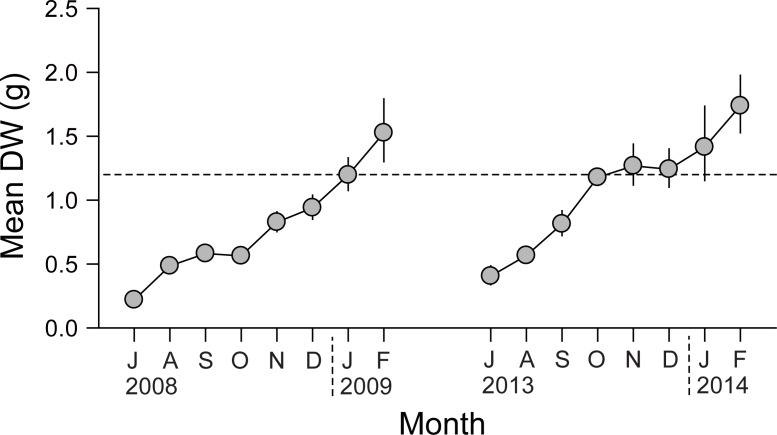
Seasonal variations in dry tissue weight (DW) of monthly mean-size individuals of *Crassostrea gigas* in Geoje–Hansan Bay. The dotted line represents the marketable value for oysters in Korea (1.2 g DW).

Monthly mean SW of the oysters varied from 2.2 ± 1.1 g (July 2008) to 36.4 ± 10.6 g (February 2009) during the first experiment, and from 2.6 ±1.7 g (July 2013) to 36.5 ± 14.0 g (February 2014) during the second experiment, with increasing trends during both experiments after deployment in their growing out area (Fig 1). When calculated using the monthly regression slopes and intercepts of DW against the SW values, monthly mean DWs ranged from 0.22 g (July 2008) to 1.53 g (February 2009) during the first experiment, and from 0.40 g (July 2013) to 1.74 g (February 2014) during the second experiment (Fig 2), with an increasing trend after deployment. The oysters reached marketable value (1.2 g for DW) in January 2009 and October 2013 during the two successive experiments, respectively. Mean DWs showed significant differences between the first (2008−2009) and second (2013−2014) experiments (paired *t*-test, *t*_7_ = −4.815; *P* = 0.002), indicating faster growth during the second experiment.

### Physiological rates

**Fig 3:**
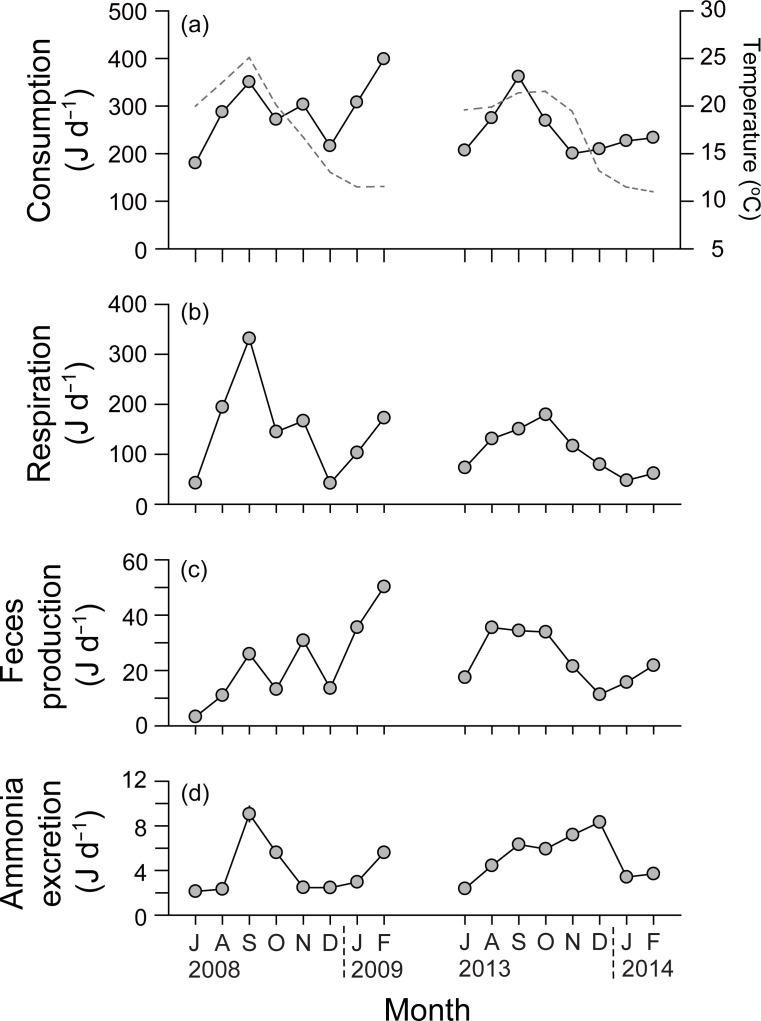
Seasonal variations in the physiological rates of monthly mean-size individuals of *Crassostrea gigas* in Geoje–Hansan Bay. (a) Consumption and temperature; (b) feces production; (c) ammonium excretion; and (d) respiration.

Individual physiological rates of monthly mean DW individuals were calculated using common slopes ($\bar{b}$) and intercepts (*ā*) of the monthly regressions of the rates against DW (Table 2), and substituting the corresponding monthly mean DWs to express as rates per monthly mean-size individual. The energy consumption of monthly mean-size individual fluctuated between 179.9 and 398.6 J d^−1^ during the first experiment, showing a seasonal pattern with high rates in summer (350.2 J d^−1^ in September 2008) and winter (398.6 J d^−1^ in February 2009) and relatively low rates in fall (215.7 J d^−1^ and 200.3 J d^−1^ in December 2008 and November 2013, respectively; Fig 3A). In contrast, energy consumption ranged from 200.4 to 361.7 J d^−1^ with a unimodal peak in September 2013 during the second experiment. Energy lost as fecal production varied from 3.2 (July 2008) to 50.3 J d^−1^ (February 2009), with irregular variation during the first experiment, and from 11.4 (December 2013) to 35.5 J d^−1^ (August 2013) during the second experiment (Fig 3B). Energy losses from excreted ammonia fluctuated from 2.1 (July 2008) to 9.1 J d^−1^ (September 2008) with a unimodal peak in summer, when water temperature was high during the first experiment, and from 2.3 (July 2013) to 8.3 J d^−1^ (December 2014) during the second experiment (Fig 3C). Metabolic energy loss by respiration also showed a clear seasonal pattern characterized by a marked peak (331.5 J d^−1^) in summer, when the water temperature was over 25.0°C during the first experiment (Fig 3D). Metabolic energy also showed an increase in summer during the second experiment, but this increase was not as high as the peak value (179.4 J d^−1^) in October 2013.

**Table 2:** Values for the intercept (*a*) and slope (*b*) in allometric equation *Y* = *aDW*^*b*^ between physiological rate (*Y*, J d^−1^) and dry tissue weight (DW, g) of *Crassostrea gigas* during experimental period.

Month	Consumption					Feces production				
	*a*	*b*	*r*	$\bar{b}$ ± 95% CI	*ā*	*a*	*b*	*r*	$\bar{b}$ ± 95% CI	*ā*
July 2008	297.3	0.431	0.890	0.444 ± 0.030	353.1	9.1	0.645	0.962	0.571 ± 0.034	7.8
August	431.0	0.333	0.787		396.1	18.6	0.842	0.776		16.7
September^**†**^	475.1	0.596	0.881		445.5	35.3	0.918	0.788		35.4
October	321.9	0.771	0.867		350.3	17.9	0.593	0.823		18.3
November	270.3	0.400	0.796		329.8	31.8	0.656	0.818		34.4
December	210.4	0.365	0.928		221.8	12.8	0.624	0.956		14.1
January 2009^**†**^	267.5	0.252	0.940		284.2	38.5	0.415	0.917		32.1
February	300.5	0.433	0.802		330.4	37.2	0.616	0.855		39.5
								
July 2013	255.8	0.370	0.841		308.9	31.0	0.364	0.811		29.3
August	304.2	0.451	0.929		353.6	48.6	0.422	0.863		49.2
September	355.1	0.471	0.804		396.5	36.8	0.416	0.725		38.7
October	309.6	0.401	0.857		250.2	34.8	0.561	0.872		30.9
November	168.0	0.535	0.837		180.3	22.4	0.711	0.804		18.8
December	178.6	0.667	0.713		190.2	10.2	0.511	0.781		10.0
January 2014	188.0	0.716	0.759		194.3	11.7	0.752	0.957		12.9
February	61.6	0.476	0.808		182.6	20.7	0.463	0.850		15.9
	Fs	df	Significance			Fs	df	Significance		
ANCOVA	1.354	15, 141	*P* = 0.182			1.313	15, 141	*P* = 0.205		
	Ammonia excretion						Respiration excretion		
July 2008	21.1	0.912	0.850	0.679 ± 0.048	6.0	145.9	0.902	0.890	0.689 ± 0.046	121.1
August	13.4	0.608	0.806		3.8	329.7	0.793	0.867		319.9
September^**†**^	46.5	0.545	0.779		13.1	359.5	0.514	0.754		441.6
October	29.3	0.437	0.903		8.3	229.4	0.798	0.749		215.3
November	9.9	0.525	0.900		2.8	179.8	0.659	0.953		190.1
December	9.1	0.611	0.777		2.6	42.4	0.743	0.906		44.1
January 2009^**†**^	9.3	0.851	0.894		2.6	85.6	1.116	0.940		91.2
February	14.9	0.789	0.777		4.2	129.5	0.777	0.743		129.1
								
July 2013	4.4	0.667	0.715		4.4	131.6	0.583	0.864		136.5
August	6.5	0.667	0.732		6.5	221.5	0.845	0.952		194.1
September	7.8	0.748	0.766		7.3	206.9	0.872	0.908		173.8
October	5.3	0.653	0.747		5.3	156.1	0.400	0.767		160.3
November	6.4	0.568	0.800		6.1	100.9	0.644	0.732		99.3
December	7.3	0.632	0.799		7.2	71.3	0.516	0.747		68.7
January 2014	2.8	0.583	0.726		2.7	44.9	0.362	0.713		37.6
February	2.6	0.586	0.742		2.6	45.0	0.496	0.863		42.2
	Fs	df	Significance			Fs	df	Significance		
ANCOVA	1.357	15, 141	*P* = 0.994			1.038	15, 141	*P* = 0.424		

Y represents consumption, respiration, feces production and ammonia excretion. Sample size (*n*) = 9 except for ^**†**^ *n* = 8 in September 2008 and January 2009. Results of ANCOVA to test significance of differences in slope are summarized at the bottom. ā, recalculated using common slopes $\bar{b}$ obtained from analysis of covariance (ANCOVA). CI, confidence interval.

### Scope for growth and net growth efficiency

**Fig 4:**
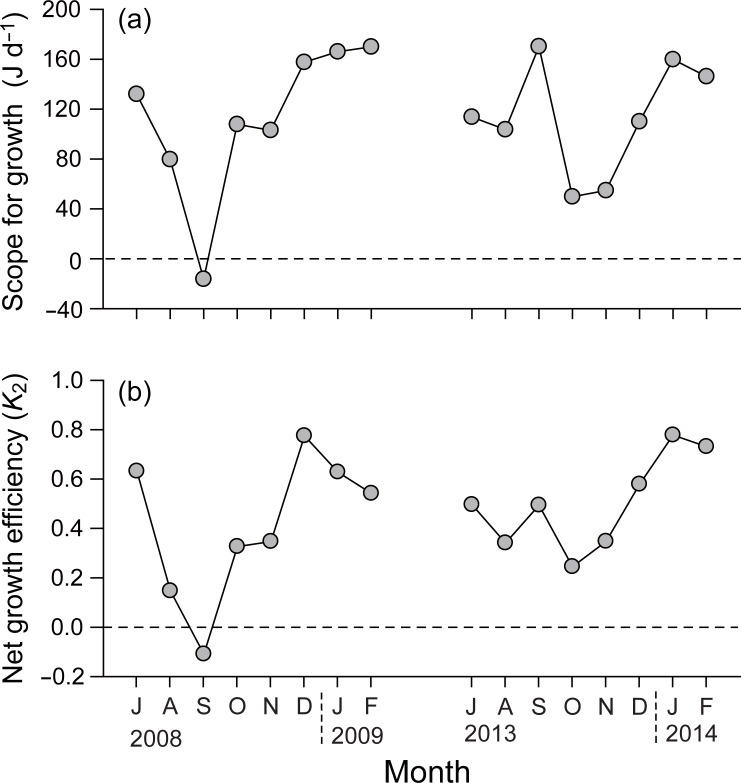
Seasonal variations in (a) scope for growth and (b) net growth efficiency of monthly mean-sized individuals of *Crassostrea gigas* in Geoje–Hansan Bay.

SFG fluctuated from −16.4 (September 2008) to 170.0 J d^−1^ (February 2009) during the first experiment, and from 49.7 (October 2013) to 170.2 J d^−1^ (September 2013) during the second experiment, displaying irregular seasonal variation (Fig 4A). No significant difference in SFG was found between the first and second experiments (paired *t*-test, *t*_7_ = −0.037; *P* = 0.971). A negative SFG value (−16.4 J d^−1^) was detected in September 2008. Net growth efficiency (*K*_2_) tended to be high in winter compared to summer, fluctuating in a similar manner to that of SFG (Fig 4B).

### Gross biochemical composition

**Fig 5:**
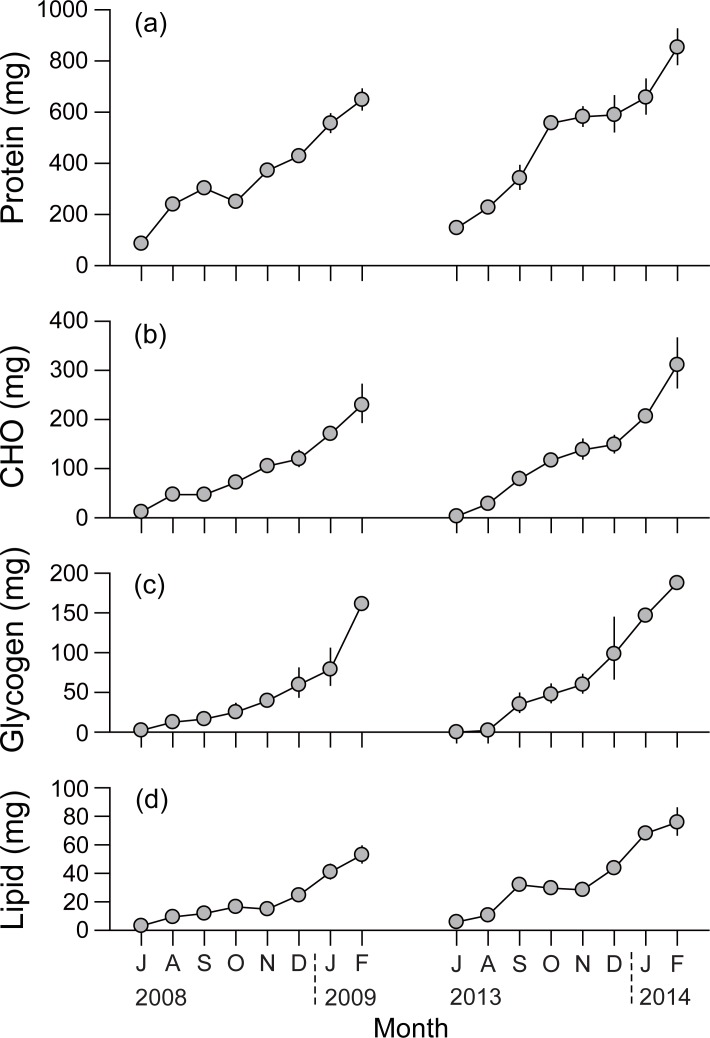
Seasonal variations in gross weights of biochemical components of monthly mean-sized individuals of *Crassostrea gigas* in Geoje–Hansan Bay. (a) Proteins; (b) carbohydrates (CHO); (c) glycogen; and (d) lipids.

**Fig 6:**
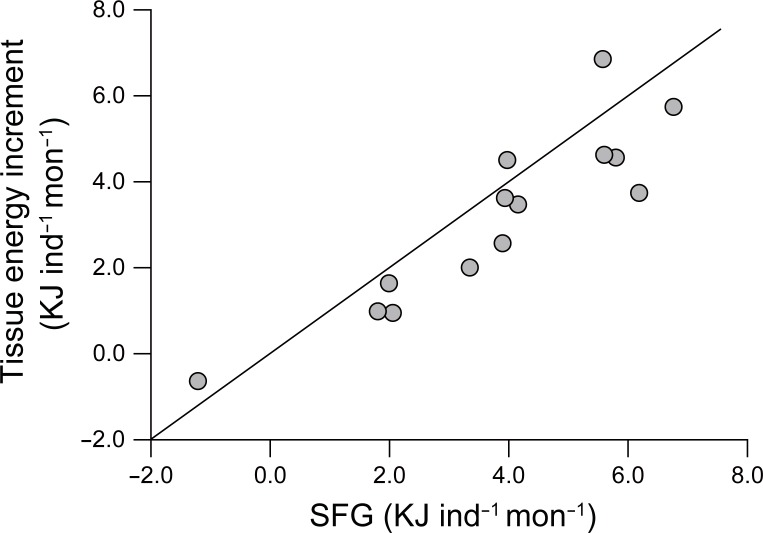
Relationship between monthly scope for growth and tissue energy increment (kJ ind^−1^ mon^−1^) of monthly mean-sized oysters. Tissue energy values were estimated by the sum of energy values of proteins, carbohydrates, and lipids. The solid line shows the linear regression and the dotted line indicates a 1:1 line.

Seasonal variations in gross biochemical composition paralleled those of DW (Fig 5), displaying a minimum in July and maximum in February for both experiments. Protein accounted for more than 50% of the whole-body tissue weight of the monthly mean-sized oysters, and varied from 85.5 to 648.1 mg during the first experiment, and from 146.7 to 852.8 mg during the second experiment (Fig 5A). Carbohydrate (glycogen) contents were 11.8–229.5 mg (2.2–161.1 mg) during the first experiment and ranged from 2.9–311.0 mg (0.0–187.4 mg) during the second experiment, corresponding to about 15% of the total tissue weight (Fig 5B and 5C). Lipid contents were 3.1–52.9 mg during the first experiment and 5.8–75.7 mg during the second experiment, contributing less than 5% to the total tissue weight (Fig 5D). Monthly increases in tissue energy values, the summation of gross values of biochemical components, displayed a close correlation with the SFG values integrated during the corresponding period (Pearson’s correlation coefficient, *r*^2^ = 0.8083, *P* < 0.001; Fig 6).

### Gross energy budget of monthly mean-size oyster individual

**Fig 7:**
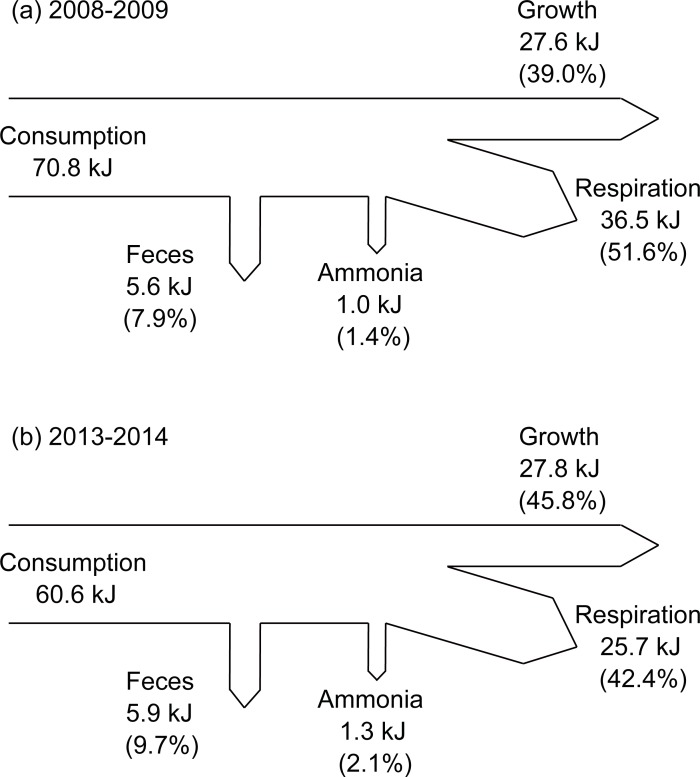
Gross energy budget of monthly mean-sized oyster individuals during the entire culturing period.

During the culturing period, the estimated energy acquired through feeding of individual mean-sized oysters from deployment to harvesting were 70.8 kJ and 60.6 kJ during the first and second experiments, respectively (Fig 7). Of these, respiration energy accounted for most of the energy losses (36.5 kJ and 25.7 kJ, respectively), corresponding to 51.6% and 42.4% of the consumed energy for the first and second experiments. Energy losses to fecal production were 5.6 kJ and 5.9 kJ, respectively, displaying 7.9% and 9.7% of the consumed energy. Energy loss values to ammonia excretion were 1.0 kJ and 1.3 kJ, respectively, explaining minor portions of 1.4% and 2.1% of the consumed energy. Flesh tissue energy production rates were finally calculated to be 27.6 kJ and 27.8 kJ, respectively, accounting for 39.0% and 45.8% of the consumed energy.

## Discussion

The total increase in mass of *C*. *gigas* over the 8-month culture period was about 1.31 and 1.33 g for the first and second experiments, respectively, corresponding to about 22.2 kJ for both experiments when converted to energy values by gross biochemical composition. Because our tissue growth estimation does not include production of the organic matter of the shell and mucus secreted [12,50], this tissue increase can be explained by our production estimations of 27.6 and 27.8 kJ from the first and second experiments, respectively, which reflect their physiological energy budget in field conditions during the entire culturing period (Fig 7). Furthermore, our field measurements of individual physiological activities for the cultured oysters during an entire culturing period enable to assess the feedback effects of oyster stocks on the bay ecosystem, acting as an ‘ecosystem engineer’ in the bay.

### Physiological processes

Individual physiological rates (i.e., food consumption, feces production, ammonia excretion, and respiration rates) of *C*. *gigas* followed positive allometry with DW (Table 2). Because the ANCOVA for individual physiological rates revealed identical slope estimates ($\bar{b}$) among the monthly regressions, weight-specific physiological rates (J g^−1^ d^−1^) of oysters can be compared by the intercepts (*ā*) of the regressions. These physiological responses to different environmental conditions on each experiment are generally reflected in patterns of short-term energy balance as well as seasonal energy reserves as illustrated in temperate marine invertebrates [28,51].

The regression exponent of 0.444 in the allometric relationship between the food consumption rate and DW in the present study is close to the values of 0.439 [38] and 0.279 [52] of *C*. *gigas*, but corresponded to the lower limit of values (0.310–0.820) for the feeding rate of suspension feeders [9,53], indicating more pronounced reduction of feeding in larger than in smaller individuals in field conditions feeding on natural SPM diets compared with those in experimental conditions feeding cultured algal diets [9,54]. These authors documented that the intercept of feeding rate is greatly influenced by diet quality and quantity, and hence is likely to change dramatically between cultured algal diets and natural diets. They also exemplified interspecific variability in filtration rates due to difference in gill area, explaining possible reduction of feeding in smaller individuals in this study. Monthly intercept estimates indicated significant seasonal variability in the daily energy acquisition rate of the oysters, and were correlated with temporal variation in thermal conditions (Pearson correlation coefficient, *r* = 0.67; Fig 3). Suspension-feeding bivalves regulate their feeding physiology (i.e., clearance, selection, ingestion, and absorption) depending on the concentration and composition of SPM [21,55,56]. Interestingly, no correlation was observed between monthly intercepts for energy use and SPM concentrations [and also *f* (%POM) values] in the present study, which indicates a lack of response (i.e., increase or decrease in individual feeding rates) to the concentration and quality of SPM. Indeed, in the present longline oyster-culturing bay system, SPM concentrations were very low and fell within a narrow range (1.6–31.3 mg l^‒1^) and *f* values were fairly constant throughout the culturing period, compared with oysters cultured in tidal flats and estuaries, where high loads and large fluctuations (up to 500 mg l^‒1^) of SPM, result in alterations in the physiological feeding mechanisms of bivalves [57,58]. Salinity in the bay varied within a narrow range of 30–34 [35] and never fell below the 20 level that decreases the oyster filtration rate [1]. Thus, our results suggest that the weight-specific feeding rates of the oysters cultured in Geoje–Hansan Bay might be regulated by seasonal variability in the ambient temperature, irrespective of food concentration and quality.

A common exponent of 0.571 of the fecal production rate–DW regressions obtained for *C*. *gigas* in the present study was close to the weight exponent of food consumption rates compared with that of other energy loss components (i.e., ammonia excretion and respiration rates). Monthly intercept estimates were very low with no clear seasonality. Pseudofeces production of *C*. *gigas* was rarely observed in our measurements under field conditions being consistently low SPM concentrations. This result suggests that most of the filtered particles are ingested and much of the ingested energy is absorbed (more than 85% of energy consumed) by oysters under low SPM loads [51] (see also Table 3). The exponents in the allometric equations between ammonia excretion rates and DW are highly variable in bivalve mollusks, depending on the relative contributions of proteins vs. carbohydrates and lipids to catabolic substrates [9]. A common weight exponent of 0.679 on the ammonia excretion rate for *C*. *gigas* in the present study was intermediate, between low (0.428) and high (1.480) limits for diverse feeding categories of mollusks [59,60,61]. No apparent correlations of monthly elevations with exogenous variables (temperature, SPM concentrations) or with endogenous ones (food consumption, respiration) were found in the present study. Our results suggest that reducing fecal production caused by high energy absorption and ammonia excretion might be an adaptive response of the oysters to maximize energy assimilation in consistently low nutritional conditions, independently of ambient temperature, feeding, and metabolic activities. It is well known that absorption efficiency and thus fecal energy loss is usually determined by diet quality [56,57,58]. Good diet quality in the Geoje-Hansan Bay, i.e. the observed high *f* (%POM) values throughout the year (Table 1), may account for high absorption efficiency in our longline oysters (Table 3).

**Table 3:** Energetic efficiency of the *Crassostrea gigas* in the Geoje-Hansan Bay.

	Definition in this study	Bay
Total area (m^2^)		5.90 × 10^7^
Oyster harvest (wet flesh weight, g)		6.73 × 10^9^
Oyster mean wet flesh weight (g ind^–1^)		7.80
Oyster density (ind m^–2^, *ρ*)		14.6
Dynamic indices (×10^3^ J m^–2^ d^–1^)		
• Ingestion per unit area (*a*)	Ingestion rate × *ρ*	3.93
• Assimilation per unit area (*b*)	(*a*—Feces production rate) × *ρ*	3.58
• Primary productivity (*c*)		42.7
• Oyster production per unit area (*d*)	Mean daily SFG × *ρ*	1.00
Efficiencies (%)		
• Exploitation efficiency (*e*)	*a* / *c*	9.21
• Assimilation efficiency (Absorption efficiency, *g*)	*b* / *a*	91.1
• Net production efficiency (Net growth efficiency, *h*)	*d* / *b*	28.0
• Gross production efficiency	*g* × *h* = *d* / *a*	25.5
• Ecological efficiency (Trophic efficiency)	*e* × *g* × *h* = *d* / *c*	2.35

The weight exponent value of 0.689 on metabolic energy cost measured as the respiration rate for *C*. *gigas* in the present study is slightly lower than those reported for the same species (0.77, [62]; 0.800, [38]), but close to the mean of 0.727 (± 0.130) obtained for suspension-and deposit-feeding mollusks [9]. A higher exponent value for the daily metabolic energy cost than for the filtration rate has generally been found in other suspension feeders [9]. On the other hand, monthly intercepts of the respiration rate–DW regressions were positively correlated with water temperature (*r* = 0.79), rather than SPM concentration, revealing a limited ability to adjust metabolic rate to increasing temperature (cf. acclimatory metabolism in *Cerastoderma edule*, [51] and *Ostrea edulis*, [63]).

### Gross energy budget of an individual during an entire culturing period

Here, monthly estimates of SFG reflected the effects of *in situ* environmental conditions on the overall performance of an individual represented by monthly mean size. This energy budget calculation showed that the SFG values of oysters were positive during most of the culturing period, suggesting favorable conditions in the bay system. One exception was noted that a negative SFG was recorded in September 2008 when the water temperature was extremely high (up to 25°C). The yearly variation in summer SFG was potentially due to slightly different feeding and metabolic responses to increasing temperatures [38]. Bougrier el al. observed maximum feeding activity of *C*. *gigas* at temperatures around 20°C and a consistent increase in its respiration over the range of temperatures (5–32°C). Our results indicate that the increase in metabolic energy losses of oysters in summer are offset by increasing energy acquisition by feeding to allow a positive energy balance, but not in extremely warm conditions. This result further suggests that a failure of metabolic adjustment of the oysters to extremely high temperatures (e.g., in September 2008) might be responsible for negative SFG and thereby the delayed growth during that time.

The consistently positive SFG values of oysters indicated the progressive accumulation of reserve materials (proteins, carbohydrates, and lipids) in their tissues during the entire culturing period, except for the slight reduction in gross protein weight in September–October 2008. Such an energy balance-related pattern of stored reserves suggests that longline-cultured oysters would possess temperature-dependent physiological adjustment processes to optimize their energy acquisition in a low nutritional (i.e., low SPM concentration) condition in the Geoje–Hansan Bay system. Our results further indicate that, despite high stock density, the adaptive response of oysters like increasing absorption [increased filtration and decreased fecal production due to high quality (*f*) of SPM] under conditions of low SPM availability enables positive production during the entire culturing period.

As indicated by seasonal trends in the DWs of the monthly mean-size oysters during the two culturing periods, oysters displayed an interannual variation in their seasonal growth patterns. Oysters reached marketable size in January 2009 in the first experiment, but in October 2013 in the second experiment (Fig 2) because oyster growth was fast at the beginning of the latter experiment, but delayed until the end of the culturing period in the former experiment. These seasonal growth patterns coincided well with the physiological rates and thereby SFG (Figs 3 and 4). While negative SFG in September of the first experiment resulted from highly elevated metabolic energy losses of oysters due to high temperatures, a highly positive SFG at the end of the culturing period (i.e., late autumn–winter) was attributed to the enhanced energy consumption and reduced metabolic losses. Therefore, metabolic responses of oysters to seasonal changes in water temperature might explain temporal patterns in their SFG and somatic growth, leading to a delay in their main growing period to winter. In contrast, such a summer cessation in tissue growth appeared to be rarely feasible because of low metabolic losses that promoted elevated SFG and fast somatic growth at the beginning of the second experiment. These physiological and thereby somatic performances can be due to the rapid accumulation of energy reserves (e.g., proteins and carbohydrates) during the periods coinciding with highly positive SFG of both culturing periods. Gross weights of lipids were slightly higher in the winter of the second than in the first culturing period, suggesting the initiation of gametogenesis in the autumn of the second period [27,64]. Indeed, gametogenesis of oysters is known to be initiated in autumn in this bay [27]. Although this gametogenic activity likely depends on previously accumulated energy reserves during the initial stage, further study is needed to test the energy reserve–gametogenesis relationship.

The seasonal patterns in physiological rates (and the resultant SFG) and gross biochemical composition (thereby DW) differed between the two culturing periods. However, net production of an oyster individual during an entire culturing period was nearly identical for the first (27.6 kJ) and second (27.8 kJ) periods (Fig 7). These SFG values were well reflected in the tissue energy increases of oysters, as indicated by their close linear relationship (Fig 6). However, our monthly estimates of tissue energy increment (kJ in d^−1^ mon^−1^) were slightly lower compared with those of SFG. Several lines of evidence are present for the gap between monthly estimates of SFG and tissue energy increases in our study. First, our estimation of tissue energy increment did not cover the organic matter of the shell and mucus secreted [12,50], as discussed above. Second, while SFG values were based on daily measured physiological rates, tissue energy increases were based on monthly measurements. Finally, slightly lower values in the estimated tissue energy might also partially result from underestimation of the protein contents in tissue and food consumption by the Lowry method compared with the metabolic energy loss values measured by oxygen probes [65].

In the present study, individual oysters displayed gross energy consumptions of 70.8 and 60.6 kJ by feeding, and a final mean tissue energy production of 27.7 kJ, corresponding to 51.6% and 42.4% of consumed energy during the entire culturing period. Previously reported tissue energy increases of 2-year-old adult oysters during the same period of January 1996 to September 1997 were about 8.5 kJ [27]. Kang et al. kept rearing the oysters with no harvesting in winter, and showed that the oyster production in the second culturing year accounted for only 30% of that of the first culturing year. Furthermore, physiological responses and gross energy budget of our submerged longline-culture oysters differed slightly from those of oysters reared using racks and bags under high SPM loads in the Marennes–Oléron Basin in France [50], which are likely to encounter much lower food quality and higher production of feces (and pseudofeces) than those of our longline oysters. This would contribute to a lower absorption efficiency. Indeed, one-year-old oysters reared on the intertidal bed consumed a total energy of 415 kJ [12]. Deslous-Paolli et al. [12] reported individual tissue energy increases by about 15 kJ (3.6% of the consumed energy; see also [66]), which corresponds to approximately half of our longline oysters. The low tissue increases resulted from high quantities of feces (as well as pseudofeces) production (315 kJ; 74% of the consumed energy) and metabolic losses (90 kJ; 21%). In contrast, energy loss by fecal production only comprised on average 8.8% of the consumed energy in our longline oysters, which is fairly low compared with the Marennes–Oléron population. This may reflect much higher food quality (Table 1) and thus absorption efficiency (Table 3) of longline oysters than of the French oysters. This substantial difference suggests that longline oysters might maximize their assimilation of consumed food under the low SPM conditions of the Geoje–Hansan Bay.

Interestingly, our estimation of tissue energy increases from June to February is much lower than the ~50 kJ recorded for longline-cultured 1-year-old oysters in Gamak Bay on the southern coast of Korea during the same period [67,68]. Such a site-dependent difference in tissue growth (1.74 g vs. 2.78 g in final DW in February) of longline oyster populations is correlated with annual primary productivity of phytoplankton in the Geoje–Hansan (9,450 t C) and Gamak (37,000 t C) Bays [29]. In addition, 20–30 kJ of tissue energy, similar to that of 1-year-old longline oysters, was achieved in the 2-year-old oysters of French intertidal beds. Consequently, our results suggest that the long-standing strategy of using submerged longline cultures and exploiting high performance of 1-year-old oysters during a short period (from summer to winter) might be acceptable as a profitable way to sustain high production in the temperate Geoje–Hansan Bay.

### Possible role as an ecosystem engineer in the bay

Cultured oysters can influence biogeochemical composition in both water and sediment of the system due to food processing and waste production [69]. Our results supported an additional attempt to quantify the feedback effects of the oyster culture on ambient coastal habitats in the context of environmentally sound and sustainable development of longline oyster cultures. The cultured oysters (*C*. *gigas*) exploit naturally occurring phytoplankton and thus influence phytoplankton communities [19,20], especially with selective feeding on diatoms [70]. Their active suspension feeding often results in the depletion of available food in the ambient water and thereby reductions in the growth rates of oyster themselves by limiting access to resources in the longline aquaculture systems [5,71]. Of a total bay area of 5.9 × 10^7^ m^2^, the oyster-culturing area accounts for 6.6 × 10^6^ m^2^, in which the oyster density deployed was a mean of 14.6 individuals m^‒2^ during the second experimental period (Table 3). During that time, 6.73 × 10^3^ t of wet oyster flesh were harvested within the whole bay. We tried to characterize the material processing performed by the cultured oysters (Table 3) [72] based on monthly physiological measurements during the entire culturing period.

Our energy budgets for oyster individuals can be utilized to quantify top-down pressure of the longline-cultured oysters on primary producers at a whole bay level. Considering the ingestion rate of individual oysters (Fig 7), the mean daily ingestion of food energy during the growth period was 3.93 kJ m^‒2^ d^‒1^ (mean 3.58 kJ m^‒2^ d^‒1^ for assimilation) at the whole bay scale, yielding a mean of 1.00 kJ m^‒2^ d^‒1^ in the daily production of oyster flesh. Taking 42.7 kJ m^‒2^ d^‒1^ (0.93 g C m^‒2^ d^‒1^) of primary productivity in the bay water column into account (unpublished data), the exploitation efficiency (amount of food ingested/amount of primary production) and assimilation efficiency (assimilation/food ingestion) were 9.21% and 91.1%, respectively, at the whole bay level, and net production efficiency by the oyster assimilation was 28.0%. This assimilation efficiency is equivalent to the upper limit values for *C*. *gigas* measured under laboratory conditions (48.3–91.1%; [73]). This value is also higher than those reported for other marine filter feeders (e.g., 42% for *Crassostrea virginica*, [74]; 45–75% for juvenile *Aplysia punctate*, [75]; and 34% for *Pyura stolonifera*, [76]). Such a high assimilation efficiency indicates that the cultured oysters likely possess an adaptive ability to maximize their utilization of high food-quality resources under relatively low SPM concentrations in the coastal marine area of the Geoje–Hansan Bay (Table 1) compared with general estuarine tidal flats. Gross production efficiency (oyster production/food ingestion) was estimated as 25.5%, which is slightly lower than the average value (33%) for marine benthic invertebrates [77]. This result represents more metabolic energy expenditure than tissue production, supporting our results of physiological measurements (Fig 7). As calculated in terms of a series of energetic efficiencies, ecological efficiency at the whole bay level corresponded to 2.4%. This estimate is similar to that for *C*. *gigas* cultured in Taiwan (2.7% recalculated from Lin et al. [78]). However, this value is lower than Lindeman’s law of trophic efficiency (10%). The low energetic efficiency values of oysters in the present study area might reject the assumption that marine suspension feeders could be an intrinsically efficient energy convertor based on their lack of energy use for thermal homeostasis and mobility [79].

Dense populations of filter-feeding bivalves can act as a provider for organic deposition to benthic systems and enhance particle flux in the form of biodeposition, representing a package or aggregation of suspended particles [11,80,81]. In our study, the cultured oyster individual produced a mean of 41.8 mg dry weight d^‒1^ of deposition, which made an estimate of a mean of 5.5 g m^‒2^ d^‒1^ (167.6 g m^‒2^ mon^‒1^) in the culturing grounds. Here, we estimated the biodeposition rate of the oysters as weight units to make comparisons with other studies. Regional comparison of biodeposition rates can result in a biased assessment because they depend upon seston concentration as well as oyster size [82]. Nevertheless, our individual-based biodeposition estimate is much lower than other estimates worldwide: 0.12–0.22 g dry weight ind^‒1^ d^‒1^ in British Columbia [11], 2.4–30 g dry weight ind^‒1^ d^‒1^ in Marennes–Oléron Bay (France) [83], and 0.06–0.29 g dry weight ind^‒1^ d^‒1^ in Southern Tasmania (Australia) [81]. The contribution of oyster biodeposition to local sediments in the present study also was low than had been measured in other locations: 35 g dry weight m^‒2^ d^‒1^ in the York estuary (Chesapeake Bay) [84], 5.1–390 g dry weight m^‒2^ d^‒1^ in the Ofunato estuary (Japan) [85], and 5.7 g dry weight m^‒2^ d^‒1^ in British Columbia [11]. As a result, the contribution of the longline oyster biodeposition was a total of 8,640 t to the bottom sediment of the whole culturing area during the entire period. Biodeposition by cultured bivalves can cause the accumulation of high masses of organic matter to the sediment, which may promote hypoxia in the bottom layer of relatively stagnant water columns like Geoje-Hansan Bay [86,87]. Indeed, lower organic contents in the bay sediment compared with those in the adjacent non-cultured bay and nearshore coastal sediments suggest a weak effect of cultured oysters on sediment properties in the bay [88]. Measurement of the decomposition rate of sedimentary organic matter is beyond the scope of the present study. No severe hypoxic events were observed throughout the year in the bottom layer of the Geoje–Hansan Bay [34,35], probably reflecting the low biodeposition rates of the oysters, as discussed above.

In addition to the release of biodeposits after feeding on suspended particles, cultured oysters excrete dissolved inorganic nutrients into the water column, leading to the rapid cycling of nutrients to stimulate phytoplankton growth [23,89]. Here, the rate of direct ammonia excretion into the water was estimated as a total of 5.3 g NH_4_ m^‒2^ (21.5 mg NH_4_ m^‒2^ d^‒1^) in the culturing ground during the entire period. Considering a total of 227 g C m^‒2^ of primary productivity in the bay during the entire culturing period (unpublished data) and the Redfield C:N ratio (~6.62), our estimated value indicates that the ammonia excreted from oysters is likely to have contributed only a limited portion (about 2%) of the nitrogen requirements for primary production. Direct excretion rates by oysters are commonly known to be lower than those released by mineralization of biodeposited and naturally deposited materials on the sediment [11,89,90]. In addition, upward flux from bottom sediments can be another important source of nutrient input [91]. Indeed, the release rate of dissolved inorganic nutrients from bottom sediments was previously estimated at a mean of 56.9 mg N m^–2^ d^–1^ in spring and 32.4 mg N m^–2^ d^–1^ in summer in Geoje–Hansan Bay, corresponding to 10–25% of daily nitrogen requirements for primary production of phytoplankton [91]. Considering that these values represent nitrogen release rates at the whole bay level, ammonia excretion of oysters within the culturing ground has a low impact compared with the mineralization of deposited materials on sediments.

## Conclusion

The scope for growth of *C*. *gigas* appeared to be positive during most of the culturing period, except for one month with extremely high temperatures (up to 25°C), indicating suitable conditions of the bay for oyster culturing throughout the year. Flesh tissue energy production during the entire culturing period was 27.6 kJ and 27.8 kJ for the first and second experiments, respectively. Directly measured tissue energy increases were closely linked with physiological estimations for flesh tissue energy production. The high assimilation rates suggest that longline-cultured oysters might adjust their physiological performance to relatively low concentrations of SPM in the bay system to optimize their energy acquisition. Such an adaptive adjustment like an increased absorption of energy and a reduced loss of metabolic and excretory energy resulted in positive production under high culturing density conditions. Our physiological measurements were also utilized to assess the feedback effects of longline-cultured oysters on the bay system. Ecological efficiency, estimated by a series of energetic efficiencies at the whole bay level, was relatively low (2.4%) compared with Lindeman’s law of trophic efficiency (10%). The cultured oyster individuals produced mean biodeposition of 5.5 g dry weight m^‒2^ d^‒1^ and ammonia excretion of 21.5 mg NH_4_ m^‒2^ d^‒1^ within the culturing grounds. Collectively, these results indicate that the cultured oysters may have only minor effects on the benthic and pelagic environments of the bay. Finally, our results suggest that the adaptive physiological performance of oysters and consequently weak feedback effects on ambient habitats should enable to facilitate sustainable longline aquaculture in the bay for a prolonged period without severe habitational deterioration.

## Supporting information

S1 Table**Values for the intercept (a) and slope (b) in allometric equation DW = aSW**^**b**^ **between dry tissue weight (DW, g) and dry shell weight (SW, g) of *Crassostrea gigas* during experimental period.** Results of ANCOVA to test significance of differences in slope are summarized at the bottom. ā, recalculated using common slopes $\bar{b}$ obtained from analysis of covariance (ANCOVA). CI, confidence interval.(PDF)

S2 Table**Values for the intercept (a) and slope (b) in allometric equation Y = aDW**^**b**^ **between gross biochemical components (Y, mg) and dry tissue weight (DW, g) of *Crassostrea gigas* during experimental period.** Y represents protein, carbohydrate, glycogen and lipid. Results of ANCOVA to test significance of differences in slope are summarized at the bottom. ā, recalculated using common slopes $\bar{b}$ obtained from analysis of covariance (ANCOVA). CI, confidence interval. Glycogen contents of most individuals analyzed were below the detection limit on July 2013.(PDF)

## References

[1] Mann R, Burreson EM, Baker PK. The Decline of the Virginia Oyster Fishery in Chesapeake Bay: Considerations for Introduction of a Non-Endemic Species, *Crassostrea gigas* (Thunberg, 1793). J Shellfish Res. 1991; 10: 379−388.

[2] Orensanz JML, Schwindt E, Pastorino G, Bortolus A., Casas G, Darrigran G, et al No longer the pristine confines of the world ocean: a survey of exotic marine species in the southwestern Atlantic. Biol Invasions. 2002; 4: 115−143.

[3] Miossec, L., Le Deuff, R‐M., and Goulletquer, P. 2009. Alien species alert: Crassostrea gigas (Pacific oyster). ICES Cooperative Research Report No. 299. 42 pp.

[4] Gosling E. Marine Bivalve Molluscs. 2nd Ed. Wiley-Blackwell, 2015. 536p.

[5] Héral M. Why carrying capacity models are useful tools for management of bivalve molluscs culture In: Dame RF, editor. Bivalve Filter Feeders in Estuarine and Coastal Ecosystem Processes. Berlin: Springer-Verlag; 1993 pp. 455–477.

[6] Dame RF, Prins TC. Bivalve carrying capacity in coastal ecosystems. Aquat Ecol. 1998; 31: 409–421.

[7] McKindsey CM, Thetmeyer H, Landry T, Silvert W. Review of recent carrying capacity models for bivalve culture and recommendations for research and management. Aquaculture. 2006; 261: 451–462.

[8] Byron C, Bengtson D, Costa-Pierce B, Calanni J. Integrating science into management: Ecological carrying capacity of bivalve shellfish aquaculture. Mar Policy. 2011; 35: 360–370.

[9] Bayne BL, Newell RC. Physiological energetics of marine mollusca In: Saleuddin ASM, Wilbur KM, editors. The Mollusca. New York: Academic press; 1983 pp. 407–515.

[10] Griffiths CL, Griffiths RJ. Bivalvia In: Pandian TJ, Vernberg FJ, editors. Animal Energetics: Bivalvia through Reptilia. New York: Academic Press; 1987 pp. 1–87.

[11] Bernard FR. Annual biodeposition and gross energy budget of mature Pacific oysters, *Crassostrea gigas*. J Fish Res Bd Can. 1974; 31: 185–190.

[12] Deslous-Paoli JM, Héral M. Energetic transfers between the juvenile oyster *Crassostrea gigas* and the potential food of the water in a bay. Haliotis. 1984; 14: 79–90.

[13] Bayne BL. The physiology of suspension feeding by bivalve molluscs: an introduction to the Plymouth “TROPHEE” workshop. J Exp Mar Biol Ecol. 1998; 219: 1–19.

[14] Bayne BL. Aspects of reproduction in bivalve mollusks In: Wiley ML, editor. Estuarine Processes. New York: Academic Press; 1976 pp. 432–448.

[15] Kooijman SALM. Population dynamics on the basis of budgets In: Metz JAJ, Diekman O, editors. The dynamics of physiologically structured populations. Berlin: Springer-Verlag; 1986 pp. 266–297.

[16] Kooijman SALM. Dynamic energy budgets in biological systems. Cambridge: Cambridge University Press; 2000.

[17] Newell RIE. Ecosystem influences of natural and cultivated populations of suspension-feeding bivalve molluscs: a review. J Shell Res. 2004; 23: 51–62.

[18] Ostroumov SA. Suspension-feeders as factors influencing water quality in aquatic ecosystems In Dame R.F, Olenin S, editors. The comparative roles of suspension-feeders in ecosystems. Dordrecht: Springer; 2005 pp. 147–164.

[19] Souchu P, Vaquer A, Collos Y, Landrein S, Deslous-Paoli JM, Bibent B. Influence of shellfish farming activities on the biogeochemical composition of the water column in Thau lagoon. Mar Ecol Prog Ser. 2001; 218: 141–152.

[20] Huang CH, Lin HJ, Huang TC, Su HM, Hung JJ. Responses of phytoplankton and periphyton to system-scale removal of oyster-culture racks from a eutrophic tropical lagoon. Mar Ecol Prog Ser. 2008; 358: 1–12.

[21] Deslous-Paoli JM, Sonin JM, Héral M. Variations saisonnières in situ de la production et de la composition des biodépots de trois mollusques estuariens (*Mytilus edulis*, *Crassostrea gigas*, *Crepidula fornicata*). Haliotis. 1987; 16: 233–245.

[22] Dame RF. The role of bivalve filter feeder material fluxes in estuarine ecosystems In: Dame RF, editor. Bivalve filter feeders in estuarine and coastal ecosystem processes. Heidelberg: Springer-Verlag; 1993 pp. 245–269.

[23] Smaal AC, Prins TC. The uptake of organic matter and the release of inorganic nutrients by bivalve suspension feeder beds In: Dame RF, editor. Bivalve filter feeders in estuarine and coastal ecosystem processes. Heidelberg: Springer-Verlag; 1993 pp. 271–298.

[24] Jones CG, Lawton JH, Shachak M. Organisms as ecosystem engineers In: Samson FB, Knopf FL, editors. Ecosystem management. New York: Springer-Verlag; 1994 pp. 130–147.

[25] Prins TC, Smaal AC, Dame RF. A review of the feedbacks between bivalve grazing and ecosystem processes. Aquat Ecol 1997; 31: 349–359.

[26] Porter ET, Cornwell JC, Sanford LP, Newell RIE. Biofiltration, water quality, and sediment processes In: Petersen JE, Kennedy VS, Dennison WC, Kemp WM. editors. Enclosed Experimental Ecosystems and Scale: Tools for Understanding and Managing Coastal Ecosystems. New York: Springer; 2009 pp. 190–194.

[27] Kang CK, Park MS, Lee PY, Choi WJ, Lee WC. Seasonal variations in condition, reproductive activity, and biochemical composition of the Pacific oyster, *Crassostrea gigas* (Thunberg), in suspended culture in two coastal bays of Korea. J Shellfish Res. 2000; 19: 771–778.

[28] Kang CK, Choy EJ, Lee WC, Kim NJ, Park HJ, Choi KS. Physiological energetics and gross biochemical composition of the ascidian *Styela clava* cultured in suspension in a temperate bay of Korea. Aquaculture. 2011; 319: 168–177.

[29] Lee BD, Kang HK, Kang YJ. Primary production in the oyster farming bay. Bull. Korean Fish Soc. 1991; 24: 39–51.

[30] Kang CK, Kim PJ, Lee WC, Lee PY. Nutrients and phytoplankton blooms in the southern coastal waters of Korea: I. The elemental composition of C, N, and P in particulate matter in the coastal bay systems. J Korean Soc Oceanogr. 1999; 34: 86–94.

[31] Deslous-Paoli JM, Lannou AM, Geairon P, Bougrier S, Raillard O, Héral M. Effects of the feeding behavior of *Crassostrea gigas* (Bivalve Molluscs) on biosedimentation of natural particulate matter. Hydrobiologia. 1992; 231: 85–91.

[32] NFRDI (National Fisheries Research and Development Institute). Annual Monitoring Report of Marine Environment around Aquaculture Area in Korea. NFRDI, Busan, Republic of Korea. 2013.

[33] NFRDI (National Fisheries Research and Development Institute). Annual Monitoring Report of Marine Environment around Aquaculture Area in Korea. NFRDI, Busan, Republic of Korea. 2014.

[34] Park YG, Kim PH, Jung YJ, Lee KJ, Kim MS, Gol KR, et al Seasonal variation of physicochemical factor and fecal pollution in the Hansan–Geojeman area, Korea. Fish Aquat Sci. 2016; 19: 110–119.

[35] Lee YJ, Kang HY, Lee WC, Kang CK. Hydrodynamic effects on growth performance of the Pacific oyster Crassostrea gigas cultured in suspension in a temperate bay on the coast of Korea. Estuar Coast. 2017; doi: 10.1007/s12237-017-0252-z

[36] Kim JH, Hong SJ, Lee WC, Kim JB, Kim HC, Kim DM. Estimation on average residence time of particulate matters in Geoje Bay using particle tracking model. J Korean Soc Mar Environ Safety. 2016; 22: 20–26.

[37] Filgueira R, Labarta U, Fernandez-Reiriz MJ. Flow-through chamber method for clearance rate measurements in bivalves: design and validation of individual chambers and mesocosm. Limnol Oceanogr Methods. 2006; 4: 284–292.

[38] Bougrier S, Geairon P, Deslous-Paoli JM, Bacher C, Jonquières G. Allometric relationships and effects of temperature on clearance and oxygen consumption rates of *Crassostrea gigas* (Thunberg). Aquacuture. 1995; 134: 143–154.

[39] Holm-Hassen O, Lorenzen CJ, Holms RW, Strickland JDH. Fluorometric determination of chlorophyll. J Cons Perm Int Explor Mer. 1965; 30: 3–15.

[40] Parsons TR, Maita Y, Lalli CM. A Manual of Chemical and Biological Methods for Seawater Analysis. Oxford: Pergamon Press; 1984.

[41] Elliot JM, Davison W. Energy equivalents of oxygen consumption in animal energetics. Oecologia. 1975; 19: 195–201. doi: 10.1007/BF00345305 28309234 10.1007/BF00345305

[42] Bougrier S, Collet B, Geairon P, Geffard O, Héral M, Deslous-Paoli JM. Respiratory time activity of the Japanese oyster *Crassostrea gigas* (Thunberg). J Exp Mar Biol Ecol. 1998; 219: 205–216.

[43] Gnaiger E. Calculation of energetic and biochemical equivalents of respiratory oxygen consumption In: Gnaiger E, Forstner H, editors. Polarographic Oxygen Sensors. Belin: Springer-Verlag; 1983 pp. 337–345 (Appendix C).

[44] Lowry OM, Roseborough NI, Farrand AL, Randall RJ. Protein measurement with folin phenol reagent. J Biol Chem. 1951; 193: 263–275. 14907713

[45] Dubois M, Gilles KA, Hamilton JK, Rebecs PA, Smith F. Colorimetric method for the determination of sugars and related substances. Anal Chem. 1956; 28: 350–356.

[46] Bligh EG, Dyer WF. A rapid method of total lipid extraction and purification. Can J Biochem Physiol. 1959; 37: 911–917. doi: 10.1139/o59-099 13671378 10.1139/o59-099

[47] Marsh JB, Weinstein DB. Simple charring method for determination of lipid. J Lipid Res. 1966; 7: 574–576. 5965305

[48] Packard GC, Boardman TJ. The misuse of ratios to scale physiological data that vary allometrically with body size In: Feder ME, Bennett AF, Burggren WW, Huey RB, editors. New directions in ecological physiology. Cambridge: Cambridge University Press; 1987 pp. 216–239.

[49] Sokal RR, Rholf FJ. Biometry: The Principles and Practice of Statistics in Biological Research. 3rd ed. New York: W.H. Freeman and Company; 1995.

[50] Héral M. Traditional oyster culture in France In: Barnabé G, Solbe JF de IB, editors. Aquaculture. Portland: Taylor & Francis; 1990; pp. 342–387.

[51] Newell RIE, Bayne BL. Seasonal changes in the physiology, reproductive condition and carbohydrate content of the cockle *Cardium* (= *Cerastoderma*) *edule* (Bivalvia: Cardiidae). Mar Biol. 1980; 56: 11–19.

[52] Kobayashi M, Hofmann EE, Powell EN, Klinck JM, Kusaka K. A population dynamics model for the Japanese oyster, *Crassostrea gigas*. Aquaculture. 1997; 149: 285–321.

[53] Winter JE. A review on the knowledge of suspension-feeding in lamellibranchiate bivalves, with special reference to artificial aquaculture systems. Aquaculture. 1978; 13: 1–33.

[54] MacDonald BA, Bricelj VM, Shumway SE. Physiology: energy acquisition and utilization In: Shumway SE, Parsons GJ, editors. Scallops: biology, ecology and aquaculture. Amsterdam: Elsevier; 2006 pp. 417–492.

[55] Navarro E, Iglesias JIP. Infaunal filter-feeding bivalves and the physiological response to short-term fluctuations in food availability and composition In: Dame RF, editor. Bivalve filter feeders in estuarines and coastal ecosystem processes. Heidelberg: Springer; 1993 pp. 25–56.

[56] Iglesias JIP, Urrutia MB, Navarro E, Ibarrola I. Measuring feeding and absorption in suspension-feeding bivalves: an appraisal of the biodeposition method. J Exp Mar Biol Ecol. 1998; 219 71–86.

[57] Barillé L, Prou J, Héral M, Razet D. Effects of high natural seston concentration on the feeding, selection, and absorption of the oyster *Crassostrea gigas* (Thunberg). J Exp Mar Biol Ecol. 1997; 212: 149–172.

[58] Navarro JM, Widdows J. Feeding physiology of *Cerastoderma edule* in response to a wide range of seston concentrations. Mar Ecol Prog Ser. 1997; 152: 175–186.

[59] Bayne BL, Scullard C. Rates of nitrogen excretion by species of *Mytilus* (bivalvia: Mollusca). J Mar Biol Assoc UK. 1977; 57: 355–369.

[60] Farías A, García-Esquivel Z, Viana MT. Physiological energetics of the green abaone, *Haliotis fulgens*, fed on a balanced diet. J Exp Mar Biol Ecol. 2003; 289: 263–276.

[61] Kang HY, Lee YJ, Choi KS, Park HJ, Yun SG, Kang CK. Combined Effects of Temperature and Seston Concentration on the Physiological Energetics of the Manila Clam *Ruditapes philippinarum*. PloS ONE. 2016; 11: e0152427 doi: 10.1371/journal.pone.0152427 27022726 10.1371/journal.pone.0152427 PMC4811571

[62] Gerdes D. The Pacific oyster *Crassostrea gigas*: Part II. Oxygen consumption of larvae and adults. Aquaculture. 1983; 31: 221–231.

[63] Haure J, Penisson C, Bougrier S, Baud JP. Influence of temperature on clearance and oxygen consumption rates of the flat oyster *Ostrea edulis*, determination of allometric coefficients. Aquaculture. 1998; 169: 211–224.

[64] Gabbott PA. Development and seasonal metabolic activities in marine mollusks In: Wilbur KM editor, The Mollusca. New York: Academic Press; 1983 pp. 165–217.

[65] Navarro E, Iglesias JIP, Larrañaga A. Interannual variation in the reproductive cycle and biochemical composition of the cockle *Cerastoderma edule* from Mundaca Estuary (Biscay, North Spain). Mar Biol. 1989; 101: 503–511.

[66] Bacher C. Modélisation de la croissance des hûitres dans le basin de Marennes-Oléron. Rapport IFREMER-CNRS. 1987. 12 p.

[67] NFRDI (National Fisheries Research and Development Institute). Annual Monitoring Report of Marine Environment around Aquaculture Area in Korea. NFRDI, Busan, Republic of Korea. 2007.

[68] Mondol MR, Kim CW, Kang CK, Park SR, Noseworthy RG, Choi KS. Growth and reproduction of early grow-out hardened juvenile Pacific oysters, *Crassostrea gigas* in Gamakman Bay, off the south coast of Korea. Aquaculture. 2016; 463: 224–233.

[69] Dumbauld BR, Ruesink JL, Rumrill SS. The ecological role of bivalve shellfish aquaculture in the estuarine environment: A review with application to oyster and clam culture in West Coast (USA) estuaries. Aquaculture. 2009; 290: 196–223.

[70] Kang CK, Choy EJ, Hur YB, Myeong JI. Isotopic evidence of particle size-dependent food partitioning in cocultured sea squirt *Halocynthia roretzi* and Pacific oyster *Crassostrea gigas*. Aquat Biol. 2009; 6: 289–302.

[71] Rodhouse PG, Roden CM. Carbon budget for a coastal inlet in relation to intensive cultivation of suspension-feeding bivalve molluscs. Mar Ecol Prog Ser. 1987; 36: 225–236.

[72] Osborne PL. Tropical Ecosystems and Ecological Concepts. Cambridge: Cambridge University Press; 2000.

[73] Gerdes D. The Pacific oyster *Crassostrea gigas*: Part I. Feeding behaviour of larvae and adults. Aquaculture. 1983; 31: 195–219.

[74] Dame RF. Energy flow in an intertidal oyster population. Estuar Coast Mar Sci. 1976; 4: 243–253.

[75] Carefoot TH. Growth and nutrition of *Aplysia punctata* feeding on a variety of marine algae. J Mar Biol Assoc UK. 1967; 47: 565–589.

[76] Klumpp DW. Nutritional ecology of the ascidian *Pyura stolonifera*: influence of body size, food quantity and quality on filter-feeding, respiration, assimilation efficiency and energy balance. Mar Ecol Prog Ser. 1984; 19: 269–284.

[77] Pomeroy LR. Secondary production mechanisms of continental shelf communities In: Livingston RJ, editor. Ecological processes in coastal and marine systems. New York: Springer; 1979 pp. 163–186.

[78] Crisp DJ. Secondary productivity in the sea In: Reichle DE, Franklin JF, Goodall DW, editors. Productivity of World Ecosystems. Washington D.C.: National Academy of Sciences; 1975 pp. 71–89.

[79] Lin HJ, Shao KT, Hsieh HL, Lo WT, Dai XX. The effects of system-scale removal of oyster-culture racks from Tapong Bay, southwestern Taiwan: model exploration and comparison with field observations. ICES J Mar Sci. 2009; 66: 797–810.

[80] Peterson BJ, Heck KL. The potential for suspension feeding bivalves to increase seagrass productivity. J Exp Mar Biol Ecol. 1999; 240: 37–52.

[81] Mitchell IM. In situ biodeposition rates of Pacific oysters (*Crassostrea gigas*) on a marine farm in Southern Tasmania (Australia). Aquaculture. 2006; 257: 194–203.

[82] Bayne BL. Oyster and the Ecosystem In: Bayne BL, editor. Biology of Oyster. New York: Academic Press; 2017 pp. 703–834.

[83] Sornin J, Feuillet M, Héral M, Deslous-Paoli JM. Effet des biodépôts de l'huître *Crassostrea gigas* (Thunberg) sur l'accumulation de matières organiques dans les parcs du bassin de Marennes-Oléron. J Molluscan Stud. 1983; 12A: 185–197.

[84] Haven DS, Morales‐Alamo R. Aspects of biodeposition by oysters and other invertebrate filter feeders. Limnol Oceanogr. 1966; 11: 487–498.

[85] Hayakawa Y, Kobayashi M, Izawa M. Sedimentation flux from mariculture of oyster (*Crassostrea gigas*) in Ofunato estuary, Japan. ICES J Mar Sci. 2001; 58: 435–444.

[86] Nizzoli D, Welsh DT, Fano EA, Viaroli P. Impact of clam and mussel farming on benthic metabolism and nitrogen cycling, with emphasis on nitrate reduction pathways. Mar Ecol Prog Ser. 2006; 315: 151−165.

[87] Hargrave BT, Doucette LI, Cranford PJ, Law BA, Milligan TG. Influence of mussel aquaculture on sediment organic enrichment in a nutrient-rich coastal embayment. Mar Ecol Prog Ser. 2008; 365: 137–149.

[88] Kang CK, Lee PY, Park JS, Kim PJ. On the distribution of organic matter in the nearshore surface sediment of Korea. Korean J Fish Aquat Sci. 1993; 26: 557–566.

[89] Smaal AC, Zurburg W. The uptake and release of suspended and dissolved material by oysters and mussels in Marennes-Oléron Bay. Aquat Living Resour. 1997; 10: 23–30.

[90] Prins TC, Smaal AC. The role of the blue mussel *Mytilus edulis* in the cycling of nutrients in the Oosterschelde estuary (The Netherlands). Hydrobiologia. 1994; 282: 413–429.

[91] NFRDI (National Fisheries Research and Development Institute). Annual report of Environmental Research of Aquaculture Farm 2008. NFRDI, Busan, Korea, 2009. 243 p

