# Supplementary material for: Physiological processes and gross energy budget of the submerged longline-cultured Pacific oyster Crassostrea gigas in a temperate bay of Korea
Source: PLoS One. 2018 Jul 5;13(7):e0199752. doi: 10.1371/journal.pone.0199752 (PMC6033403; doi:10.1371/journal.pone.0199752)
Supplement: S1 Table — Values for the intercept (a) and slope (b) in allometric equation DW = aSWb between dry tissue weight (DW, g) and dry shell weight (SW, g) of Crassostrea gigas during experimental period. Results of ANCOVA to test significance of differences in slope are summarized at the bottom. ā, recalculated using common slopes b¯ obtained from analysis of covariance (ANCOVA). CI, confidence interval. (PDF) [file pone.0199752.s001.pdf]

Month	a	b	*r*	$\bar{b}$ ± 95% CI	ā
Jul 2008	0.117	0.809	0.772		
Aug	0.047	1.152	0.832		
Sep	0.104	0.685	0.751		
Oct	0.007	1.416	0.900		
Nov	0.040	0.905	0.730		
Dec	0.054	0.889	0.721		
Jan 2009	0.017	1.249	0.767		
Feb	0.018	1.232	0.889		
Jul 2013	0.135	0.9116	0.802		0.121
Aug	0.081	1.1451	0.880		0.103
Sep	0.108	0.9483	0.746		0.097
Oct	0.056	1.1245	0.904	1.002 ± 0.052	0.078
Nov	0.049	1.0134	0.806		0.051
Dec	0.044	1.0623	0.846		0.053
Jan 2014	0.071	0.8543	0.796		0.042
Feb	0.039	1.0494	0.825		0.046

ANCOVA	Fs	df	Significance
All	2.080	15, 396	*P* = 0.010
2008-2009	3.440	7, 221	*P* = 0.002
2013-2014	0.468	7,175	*P* = 0.857
