# Supplementary material for: Physiological processes and gross energy budget of the submerged longline-cultured Pacific oyster Crassostrea gigas in a temperate bay of Korea
Source: PLoS One. 2018 Jul 5;13(7):e0199752. doi: 10.1371/journal.pone.0199752 (PMC6033403; doi:10.1371/journal.pone.0199752)
Supplement: S2 Table — Values for the intercept (a) and slope (b) in allometric equation Y = aDWb between gross biochemical components (Y, mg) and dry tissue weight (DW, g) of Crassostrea gigas during experimental period. Y represents protein, carbohydrate, glycogen and lipid. Results of ANCOVA to test significance of differences in slope are summarized at the bottom. ā, recalculated using common slopes b¯ obtained from analysis of covariance (ANCOVA). CI, confidence interval. Glycogen contents of most individuals analyzed were below the detection limit on July 2013. (PDF) [file pone.0199752.s002.pdf]

Month	Protein					Lipid				
	a	b	r	$\bar{b}$ ± 95% CI	ā	a	b	r	$\bar{b}$ ± 95% CI	ā
Jul 2008	403.4	1.021	0.921			15.6	1.075	0.821		15.3
Aug	482.2	0.971	0.974			19.9	1.051	0.924		20.1
Sep	490.1	0.891	0.957			23.7	1.296	0.834		20.9
Oct	397.4	0.812	0.970			30.9	1.101	0.976		30.2
Nov	449.1	0.995	0.978			17.7	0.932	0.965		18.2
Dec	449.1	0.777	0.986			26.4	1.162	0.983		26.2
Jan 2009	473.2	0.899	0.957			33.3	1.152	0.943		33.8
Feb	438.4	0.925	0.952			34.5	1.008	0.887		33.8
									1.061 ± 0.027	
Jul 2013	432.0	1.194	0.959			13.1	0.892	0.719		15.2
Aug	382.2	0.918	0.958			21.2	1.235	0.915		19.2
Sep	421.8	1.017	0.817			38.8	0.945	0.884		39.7
Oct	501.0	0.644	0.891			25.5	0.904	0.844		24.8
Nov	479.7	0.811	0.942			21.8	1.123	0.936		22.1
Dec	476.9	0.977	0.880			34.6	1.066	0.944		34.6
Jan 2014	466.0	0.992	0.903			48.5	0.975	0.941		47.1
Feb	498.4	0.973	0.931			44.1	0.980	0.869		42.2
ANCOVA	Fs	df	Significance			Fs	df	Significance		
All	3.739	15, 310	P = 0.000			0.798	15,307	P = 0.680		
2008−2009	5.445	7, 152	P = 0.000							
2013−2014	3.284	7, 158	P = 0.003							

Month	Carbohydrate					Glycogen		
Jul 2008	84.3	1.297	0.808		102.8	36.7	1.852	0.901
Aug	109.5	1.161	0.862		132.9	45.7	1.792	0.815
Sep	87.2	1.139	0.818		102	38.5	1.576	0.845
Oct	145.6	1.230	0.946		163.1	65.1	1.658	0.774
Nov	132.5	1.212	0.965		138	55.9	1.841	0.94
Dec	132.5	1.644	0.967		130.7	69.3	2.405	0.928
Jan 2009	132.5	1.415	0.943		132.2	55.6	1.951	0.828
Feb	132.5	1.300	0.853		125.5	95.3	1.242	0.716
				1.428 ± 0.061				
Jul 2013	16.2	1.915	0.768		10.4	-	-	-
Aug	54.6	1.155	0.759		63.8	3.1	0.784	0.924
Sep	100.3	1.169	0.758		105.8	48.5	1.585	0.828
Oct	96.6	1.152	0.878		92.4	39.2	1.165	0.886
Nov	100.6	1.341	0.884		98.6	39.7	1.725	0.876
Dec	123.0	0.884	0.850		109.3	60	2.28	0.8
Jan 2014	143.0	1.059	0.868		125.8	76.3	1.881	0.818
Feb	171.8	1.075	0.828		141.4	62.5	1.989	0.886
ANCOVA	Fs	df	Significance			Fs	df	Significance
All	1.556	15, 307	P = 0.085			3.507	15, 304	P = 0.000
2008−2009						4.674	7, 150	P = 0.000
2013−2014						4.496	7, 132	P = 0.000
